# Trajectories of adaptive functioning from early childhood to adolescence in autism: Identifying turning points and key correlates of chronogeneity

**DOI:** 10.1002/jcv2.12212

**Published:** 2023-12-14

**Authors:** Yun‐Ju Chen, Eric Duku, Peter Szatmari, Mackenzie Salt, Isabel Smith, Annie Richard, Lonnie Zwaigenbaum, Tracy Vaillancourt, Anat Zaidman‐Zait, Terry Bennett, Mayada Elsabbagh, Connor Kerns, Stelios Georgiades

**Affiliations:** ^1^ McMaster University Hamilton ON Canada; ^2^ Centre for Addiction and Mental Health The Hospital for Sick Children University of Toronto Toronto ON Canada; ^3^ Autism Alliance of Canada Toronto ON Canada; ^4^ Dalhousie University Halifax NS Canada; ^5^ Autism Research Centre IWK Health Centre Halifax NS Canada; ^6^ University of Alberta Edmonton AB Canada; ^7^ University of Ottawa Ottawa ON Canada; ^8^ Tel Aviv University Tel Aviv Israel; ^9^ McGill University Montreal QC Canada; ^10^ University of British Columbia Vancouver BC Canada

**Keywords:** adaptive functioning, autism, development, longitudinal studies

## Abstract

**Background:**

Previous research has demonstrated heterogeneous adaptive outcomes across the autism spectrum; however, the current literature remains limited in elucidating turning points and associated factors for longitudinal variability (chronogeneity). To address these empirical gaps, we aimed to provide a finer‐grained characterization of trajectories of adaptive functioning from early childhood to adolescence in autism.

**Methods:**

Our sample (*N* = 406) was drawn from an inception cohort of children diagnosed Autistic at ages 2–5. Adaptive functioning was assessed with Vineland Adaptive Behavior Scales (VABS, 2^nd^ Edition) across 6 visits from the time of diagnosis by age 18. Parallel‐process latent growth curve modeling were used to estimate domain‐level VABS trajectories, followed by latent class growth analysis to identify trajectory subgroups. Child characteristics at diagnosis, family demographics, and participation outcomes at adolescence were compared across subgroups.

**Results:**

Piecewise latent growth models best described VABS trajectories with two turning points identified at around ages 5‐6 and 9–10, respectively reflecting transitions into school age and early adolescence. We parsed four VABS trajectory subgroups that vary by level of functioning and change rate for certain domains and periods. Around 16% of the sample exhibited overall adequate functioning (standard score >85) with notable early growth and social adaptation during adolescence. About 21% showed low adaptive functioning (standard score ≤70), with decreasing slopes by age 6 followed by improvements in communication and daily‐living skills by age 10. The other two subgroups (63% in total) were characterized by adaptive functioning between low and adequate levels, with relatively stable trajectories entering school age. These subgroups differed most in their cognitive ability at diagnosis, household income, and social participation in adolescence.

**Conclusions:**

We identified key individual and family characteristics and time windows associated with distinct adaptive functioning trajectories, which have important implications for providing timely and tailored supports to Autistic people across developmental stages.

## Graphical abstract

This study examined adaptive functioning trajectories in autism from early childhood to adolescence. Two turning points were identified at ages 5‐6 and 9–10, corresponding to transitions into school age and early adolescence. Four subgroups based on these trajectories were associated with cognitive ability at diagnosis, household income, and social participation in adolescence, highlighting the importance of tailored support across developmental stages for Autistic individuals.

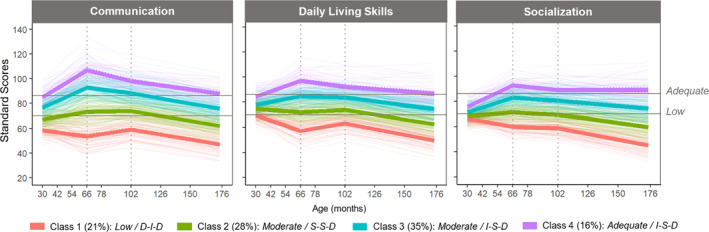

**Key points**

- Previous research is limited in clarifying *when* (e.g., turning points) and *what* beyond individual factors are associated with diverse trajectories of adaptive functioning in autism.
- Two turning points were observed at around ages 5–6 and 9–10, reflecting opportunities for improvement or additional challenges in adaptive functioning.
- Four developmental profiles of adaptive functioning with different levels and change patterns from preschool age to adolescence were identified among 406 Autistic children.
- Children classified to these profiles differed in individual and family characteristics, including cognitive ability at diagnosis, household income, and social participation in adolescence.
- Our findings highlight the importance of offering customized support to Autistic people to help them succeed at different stages of life.

## INTRODUCTION

Autism is a neurodevelopmental condition characterized by early emerging challenges in social communication and restricted/repetitive behavior that are heterogeneously manifested over time. These core autism features coupled with co‐occurring health problems may pose challenges to a child's and family's daily functioning and well‐being across the lifespan (Lord et al., 2022; Oakley et al., 2021). Despite these life‐long challenges, previous evidence suggests at least a subpopulation of Autistic people diagnosed in childhood may no longer meet diagnostic criteria for autism or may exhibit improvements in adaptive and language outcomes later in life (Anderson et al., 2014; Fein et al., 2013; Lord et al., 2015; Szatmari et al., 2021). The presence of Autistic people who move toward functional independence and fruitful lives underscores the importance of tracking longitudinal trajectories or “chronogeneity” (i.e., longitudinal heterogeneity or heterogeneity over time) to better understand the developmental process toward achieving various long‐term outcomes (Georgiades et al., 2017; Georgiades & Kasari, 2018; Lai & Szatmari, 2019). This likely involves understanding a dynamic interaction among individual, contextual, and socio‐ecological dimensions (Eigsti et al., 2022; Elsabbagh, 2020; Lai et al., 2020; Ungar, 2015), and profiles of strengths and difficulties that can change with contextual demands and supports across development (Georgiades & Kasari, 2018; Lord et al., 2022; Szatmari et al., 2016).

Adaptive functioning has been considered a clinically meaningful indicator of developmental outcomes in autism (Farmer et al., 2018; Kanne et al., 2011), which resonates with stakeholders (e.g., Autistic self‐advocates and families) as well because of the emphasis on what a person can do (Gentles et al., 2023; Tesfaye et al., 2019). Prospective studies have demonstrated heterogeneous trajectories of adaptive functioning among Autistic people characterized by various levels of functioning and change rates (Baghdadli et al., 2018; Bal et al., 2015; Bussu et al., 2019; Farmer et al., 2018; Meyer et al., 2018; Sacrey et al., 2019; Szatmari et al., 2015). Despite methodological and sampling differences across these studies, a subgroup of Autistic people with higher levels of adaptive functioning and improving or stable trajectories has been consistently identified in 15%–30% of participants. One major interest in such studies is to identify individual characteristics associated with distinct trajectories (Baghdadli et al., 2018; Georgiades et al., 2017; Pender et al., 2020). Higher language and cognitive skills during early childhood are commonly reported to be linked to improving trajectories of core autism features among Autistic people (Baghdadli et al., 2018; Bal et al., 2015; Szatmari et al., 2015). However, discrepancies among cognitive, adaptive skills, and core autism features have been observed across developmental stages and seemed to widen with age (Alvares et al., 2020; Bradshaw et al., 2019; Farmer et al., 2018; Hill et al., 2015; Kanne et al., 2011; Tillmann et al., 2019). For instance, individuals with IQ ≥ 70 do not always show adaptive functioning superior to those with IQ < 70 later in life (Lord et al., 2015; McCauley et al., 2020). Autistic adolescents with average IQ tended to show age‐appropriate daily‐living skills regardless of their level of autistic traits (Duncan & Bishop, 2015) in contrast to the stronger IQ‐symptom connections in early childhood (Di Rezze et al., 2019; Green & Carter, 2014). These findings highlight the multivariate and developmental nature of autism such that an adaptive outcome may be present for one behavioral domain but not another and their relationship can also change over time (Georgiades & Kasari, 2018; Szatmari et al., 2021).

Despite this accumulating evidence, several empirical gaps should be addressed. One is the lack of evidence on the associations between family characteristics and Autistic children's adaptive functioning trajectories. Previous research has documented links between family demographics (e.g., household income, parent education, ethnic background, and immigrant status) and early identification and diagnosis of autism (Daniels & Mandell, 2014; Thomas et al., 2012), access to and utilization of services (Khanlou et al., 2017; Lim et al., 2021; Pickard & Ingersoll, 2016; Smith et al., 2020), as well as educational placement (Gindi, 2020; Kurth et al., 2016). These factors may further influence Autistic children's adaptive outcomes (Aishworiya et al., 2021; Hodge et al., 2021; St. John & Ausderau, 2021). It is considered important to account for multiple demographic indicators when studying behavioral manifestation of autism to mitigate their confounding effects on the primary factors of interest (Durkin et al., 2017; Rai et al., 2012). Thus, examining family demographic characteristics as potential correlates of adaptive functioning trajectories could contribute to a better understanding of family roles in supporting Autistic children to reach more adaptive outcomes given the environmental demands (Lai et al., 2020; Lai & Szatmari, 2019).

Another gap lies in identifying potential turning points along the trajectories, which may reflect important life transitions (e.g., transitions to school, adolescence, and adulthood) associated with additional challenges and needs, or opportunities for change (Cicchetti & Rogosch, 2002; Georgiades et al., 2022). Despite the clinical significance of identifying critical turning points for informing support or service efforts, it remains understudied in the field of autism research. Previous research has found a turning point during the period of transition to school followed by different rates of change in core autistic traits (Georgiades et al., 2022) and nonlinear (mostly quadratic) trends in adaptive functioning development across life stages among Autistic populations (Bal et al., 2015; Bussu et al., 2019; Farmer et al., 2018; Meyer et al., 2018; Sacrey et al., 2019; Szatmari et al., 2015). Thus, further investigations are warranted to identify meaningful turning points along the developmental pathways of adaptive functioning.

The current study focused on *subdomain‐level* instead of a composite measure of adaptive functioning, as research has found that the latter may obscure domain‐specific sources of variance (Farmer et al., 2022; Waizbard‐Bartov & Miller, 2022). We also examined to what extent longitudinal heterogeneity of adaptive functioning is explained by child characteristics at diagnosis and family demographics. We further included daily activity participation (e.g., how often children take part in important daily activities, such as doing homework and spending time with peers) as a distal outcome to capture *whether a child actually takes part in daily life settings* (Estes et al., 2019; Kramer et al., 2012) to obtain a more comprehensive picture of functional performance in adolescence and to identify areas of life where individuals may require additional support based on their individual functioning. Our specific research questions were:

1. What is the best‐fitting shape of the latent growth trajectories of adaptive functioning domain‐level standard scores as measured by the Vineland Adaptive Behavior Scales (VABS) (communication, daily living, and socialization skills)? Are there turning points at certain ages?
2. How many subgroups based on adaptive functioning trajectories can be identified? Are child characteristics (sex assigned at birth, age of diagnosis, nonverbal IQ, and core autistic traits at diagnosis) and family demographics (household income, primary caregiver's education, ethnicity, and nativity) associated with subgroup membership?
3. Do these adaptive functioning trajectory subgroups differ by participation outcomes in adolescence (frequency of participating in daily activities across home, school, and community settings)?

## METHOD

### Participants and procedures

The current sample (*N* = 406) was drawn from a large Canadian prospective study (*Pathways in ASD;* see Szatmari et al., 2015, for inclusion criteria), through which 421 children and families were recruited at the time of autism diagnosis between ages 2 and 5 years across five sites (Halifax, Montreal, Hamilton, Edmonton, and Vancouver). Children were followed up with repeated assessments across childhood and adolescence. The study used an accelerated longitudinal design for the first three visits during preschool age: study enrollment (within 4 months of diagnosis), 6 months, and 12 months after enrollment. Subsequent data collection occurred at approximately ages 6, 8, 9, 10, 11, 13, and 15 years, varying by measure (see supplementary Table S1 for the repeated measures at each of the 10 visits).

### Measures

#### Adaptive functioning (main trajectory indicators)

The VABS, Second Edition (VABS‐II; Sparrow et al., 2005) is a semi‐structured parent interview that assesses children's adaptive behavior in the domains of Communication (COM), Socialization (SOC), and Daily Living Skills (DLS). We elected to use standard scores (*M* = 100, SD = 15) in the COM, SOC, and DLS domains for modeling changes in the current study, given their superior measurement properties over age‐equivalents for studying change (Tinsley, 2011). Data obtained across a total of 6 visits between ages 2 and 17 years were restructured by age for modeling age‐based trajectories. The current sample includes participants with VABS data at ≥ one visit (*N* = 406).

#### Family demographics (covariates)

Information about families' annual household income, primary caregiver's education level, ethnicity, and country of birth were gathered via the Family Background Information Questionnaire developed for the *Pathways in ASD* study based on the National Longitudinal Survey of Children and Youth (NLSCY; Statistics Canada, 2007). The household income variable comprised 11 ordinal categories ranging from less than CAN$5000 to more than CAN$80,000; the highest household income reported over the study period was used in the analysis. The primary caregiver education variable was also based on the highest level reported across the study period, recoded into three categories (1 = high school or less; 2 = some post‐secondary education; 3 = bachelor's degree or higher). The caregiver's ethnicity variable comprised 12 categories and was recoded to binary format (1 = White, 0 = Non‐White) for analysis. The primary caregiver's country of birth was coded in binary format (1 = Canada‐born, 0 = foreign‐born) from descriptive data.

#### Nonverbal cognitive ability (covariate)

Merrill‐Palmer‐Revised Scales of Development (M‐P‐R) is a standardized measure that assesses the cognitive ability of young children aged 1–78 months with minimal reliance on children's language skills (Roid & Sampers, 2004). The M‐P‐R Cognitive domain standard score (*M* = 100, SD = 15, range = 10–160) measured at study enrollment was used as a metric of nonverbal intelligence quotient (NVIQ) (Dempsey et al., 2020) in this study.

#### Core autism features (covariate)

The Autism Diagnostic Observation Schedule (ADOS; Lord et al., 2000) was administered and scored by research‐reliable examiners. The standardized total calibrated severity scores at study enrollment were used as a metric of core autism features in this study.

#### Daily activity participation (associated distal outcomes)

The Participation and Environment Measure for Children and Youth (PEM‐CY; Coster et al., 2011) is a parent‐report questionnaire that measures child's participation across 25 types of daily activities at home, school, and community. The PEM‐CY has been validated on children aged 5–17 years with typical development or developmental/physical disabilities (Coster et al., 2011). This study focused on the PEM‐CY frequency scale assessed twice between ages 12 and 17 years, where caregivers were asked to rate how often their child participated in each of the activities over the past 4 months (0 = never to 7 = daily). The internal consistency of the frequency scales derived from the current sample was low (*α* = 0.52 to 0.68; Chen et al., 2023), indicating that the items should be analyzed individually rather than aggregated into summary scores. The higher of the item‐level scores across the two time‐points were used as distal outcome variables for further analysis.

### Statistical analysis

The VABS data collected at the first three visits (ages 2–6 years) under the accelerated longitudinal design were restructured by the child's chronological age (see Table S2 for descriptive statistics of the restructured data). This resulted in a missing‐at‐random (MAR) pattern due to study design that allows for yielding unbiased parameter estimates under full information maximum likelihood (FIML) estimation (Enders & Bandalos, 2001). As the missingness due to data restructuring by age depends on the child's age of study enrollment, *age at the first visit* was included as an auxiliary variable for facilitating FIML estimation in the latent growth curve models (LGM) (Graham, 2003). First, univariate LGMs with different functional forms (linear, quadratic, and piecewise) were estimated to identify the best‐fitting trajectory per VABS domain using standard scores. In the case of piecewise LGMs, freely estimated time‐specific factor loadings, modification indices, along with visual inspection of estimated trajectories were used to detect potential turning points or knots (Kwok et al., 2010). Chi‐square statistics, comparative Fit Index (CFI), Tucker‐Lewis Index (TLI), and the root mean square error of approximation (RMSEA) were used to compare and select the best‐fitting models.

As we were interested in identifying subgroups based on the *co‐development* of the three VABS domains, a parallel‐process LGM with the best‐fitting functional form across the univariate models was further fitted for the estimation of multivariate growth. Next, latent class growth analysis (LCGA), a special type of growth mixture modeling approach where the within‐group growth factor variance is constrained to zero (i.e., assuming within‐subgroup homogeneity), was performed with the parallel‐process LGM specification to identify distinct VABS trajectory subgroups. Parallel‐process LCGAs with various numbers of classes were fitted; the optimal class solution was determined based on a set of fit indices, including Bayesian information criterion (BIC), sample size‐adjusted Bayesian information criterion (SABIC), entropy, and Lo‐Mendell‐Rubin likelihood ratio test (LMR‐LRT) statistics. Finally, the associations between adaptive functioning subgroup membership and covariates (child's sex assigned at birth, age at diagnosis, NVIQ, ADOS total‐CSS, household income, primary caregiver's education, ethnicity, and nativity), as well as distal outcomes (frequency of participation in 25 PEM‐CY activities) were examined with the three‐step approach to obtain regression and chi‐square estimates adjusted for classification uncertainty (Bakk et al., 2013).

## RESULTS

### Missing data

Among the 406 participants with VABS data from at least one of six visits, 287 (70.7%) had at least four completed assessments. Those who had ≥4 assessments did not differ from the full sample with ≥1 assessment across all child and family characteristics. The missing rates of sample characteristic variables were low (0%–5.7%) (see Table 1 for demographics across subsamples with different numbers of timepoints available). After restructuring data by age, missing data rates for each time‐point ranged from 30.0% to 77.1% for VABS. The missingness was handled by applying FIML under MAR, which can derive robust estimates from longitudinal data with missingness rates as high as ∼75% (Newman, 2003). To ensure the robustness of our latent growth estimates, we also performed sensitivity analyses under the final parallel‐process LGM specification on samples with different numbers of time‐points available. The results in Table S3 indicated no salient differences beyond median standard errors in the individual estimates of latent growth parameters derived from the parallel‐process models across data conditions.

**TABLE 1:** Sample characteristics.

	Missing rate	VABS assessed ≥1 visit (*N* = 406)	VABS assessed ≥4 visits (*N* = 287)	Samples ≥4 versus ≥1 visit(s)*t*‐test statistic (*p*)
		Mean (SD)		*t*‐test statistic (*p* value)
Age at diagnosis (months)	0%	38.35 (8.74)	38.91 (8.71)	0.83 (0.41)
NVIQ at diagnosis	4.7%	56.39 (26.82)	57.51 (26.45)	0.53 (0.59)
ADOS‐total CSS at diagnosis	0.7%	7.57 (1.71)	7.66 (1.72)	0.71 (0.48)

		N (%)		Odds ratio (*p* value)
Male	0%	342 (84.2)	246 (85.7)	1.12 (0.59)
Site				
Halifax	0%	53 (13.0)	42 (14.6)	1.14 (0.55)
Montreal		133 (32.8)	103 (35.9)	1.15 (0.39)
Hamilton		67 (16.5)	39 (13.6)	0.80 (0.29)
Vancouver		90 (22.2)	62 (21.6)	0.97 (0.86)
Edmonton		63 (15.5)	41 (14.3)	0.91 (0.65)
Family income (averaged across visits)				
Less than CAN$40,000	5.2%	70 (17.2)	41 (14.3)	0.80 (0.30)
CAN$40,000–80,000		106 (26.1)	73 (25.4)	0.97 (0.84)
CAN$80,000 or more		209 (51.5)	167 (58.2)	1.31 (0.08)
Primary caregiver's education (highest across visits)				
High school or less	4.4%	35 (8.6)	17 (5.9)	0.67 (0.19)
Some post‐secondary education		168 (41.4)	127 (44.3)	1.12 (0.45)
Bachelor's degree or higher		185 (45.6)	138 (48.1)	1.11 (0.08)
Primary caregiver's ethnicity				
White	5.7%	282 (69.5)	203 (70.7)	1.06 (0.72)
South/Southeast Asian		28 (6.9)	20 (7.0)	1.01 (0.97)
East Asian		20 (4.9)	18 (6.3)	1.29 (0.44)
Arab		16 (3.9)	10 (3.5)	0.88 (0.76)
Black		14 (3.4)	9 (3.1)	0.91 (0.82)
Indigenous		9 (2.2)	4 (1.4)	0.62 (0.44)
Latinx		7 (1.7)	7 (2.4)	1.43 (0.51)
Other		7 (1.7)	6 (2.1)	1.22 (0.73)
Primary caregiver's nativity				
Canada‐Born	5.7%	278 (68.5)	200 (69.7)	1.06 (0.73)
Foreign‐born		110 (27.1)	77 (26.8)	0.99 (0.94)

*Note*: Nonverbal IQ (NVIQ) was measured by Merrill‐Palmer‐Revised Scales cognitive domain standard score.

Abbreviation: ADOS‐total CSS, Autism Diagnostic Observation Schedule total calibrated severity score.

### Functional form and mean trajectories of VABS domains

The fit statistics of univariate LGMs with different functional forms (see Table S4) revealed that the piecewise growth models best described the observed data with knots identified at around 5–6 and 9–10 years of age (T4 and T6) across the three VABS domains (χ^2^(30) = 48.55 to 76.42, CFI = 0.976 to 0.987, TLI = 0.972 to 0.984, RMSEA = 0.039 to 0.062). A parallel‐process LGM was further fitted with the piecewise functional form, which indicated good model fit (χ^2^(267) = 485.43, CFI = 0.968, TLI = 0.959, RMSEA = 0.045).

The final piecewise model estimates (shown in Table 2) indicated that the mean trajectories of VABS Communication and Socialization were characterized by notable increases by age 6 (M_slope1_ = 3.36 & 2.30, SE = 0.45 & 0.35, *p* < 0.001), followed by decreases by age 10 (M_slope2_ = −0.68 & −0.79, SE = 0.25 & 0.35, *p* < 0.001); in contrast, standard scores in the domain of DLS remained stable by age 10 (M_slope1&2_ = 0.51 & 0.13, SE = 0.40 &. 22, *p* > 0.05). After around age 10, all the three VABS domains showed mean‐level decreases (M_slope3_ = −2.00 to −1.35, SE = 0.11 to 0.13, *p* < 0.001). Significant variances were observed for intercepts and slopes across domains (all *p* < 0.01) especially during the preschool period, with variances of slopes decreasing (i.e., change rates became less heterogeneous) over time. As expected, the growth parameters across the three domains were highly correlated (*r* = 0.58 to 0.95; Table S5), thus providing evidence of their co‐development. However, relatively low correlations (*r* ≤ 0.76) were observed between the slopes of Socialization and other domains in the second and third phases of trajectories (i.e., after the first turning point at around age 6), indicating that growth in the Socialization domain became relatively “uncoupled” from the other two domains over time.

**TABLE 2:** Piecewise latent growth parameter estimates (unstandardized) and standard errors of Vineland Adaptive Behavior Scales (VABS) domains.

	Communication		Daily living skills		Socialization	
	Mean	Variance	Mean	Variance	Mean	Variance
INT	71.64*** (1.05)	141.91*** (17.28)	76.08*** (0.88)	68.22*** (12.70)	69.75*** (0.71)	53.97*** (8.67)
SLP_1_ (2–6 years)	3.36*** (0.45)	22.47*** (3.73)	0.51 (0.40)	14.66*** (3.17)	2.30*** (0.35)	15.74*** (2.49)
SLP_2_ (6–10 years)	−0.68** (0.25)	8.33*** (1.50)	0.13 (0.22)	4.03*** (1.07)	−0.79*** (0.21)	4.18*** (1.12)
SLP_3_ (10–16 years)	−2.00*** (0.11)	1.26** (0.48)	−1.73*** (0.12)	1.47** (0.48)	−1.35*** (0.13)	2.67*** (0.65)

Abbreviations: INT, intercept; SLP, slope.

*p < 0.05, **p < 0.01, ***p < 0.001 (significantly different from 0).

### VABS trajectory classes

**FIGURE 1:**
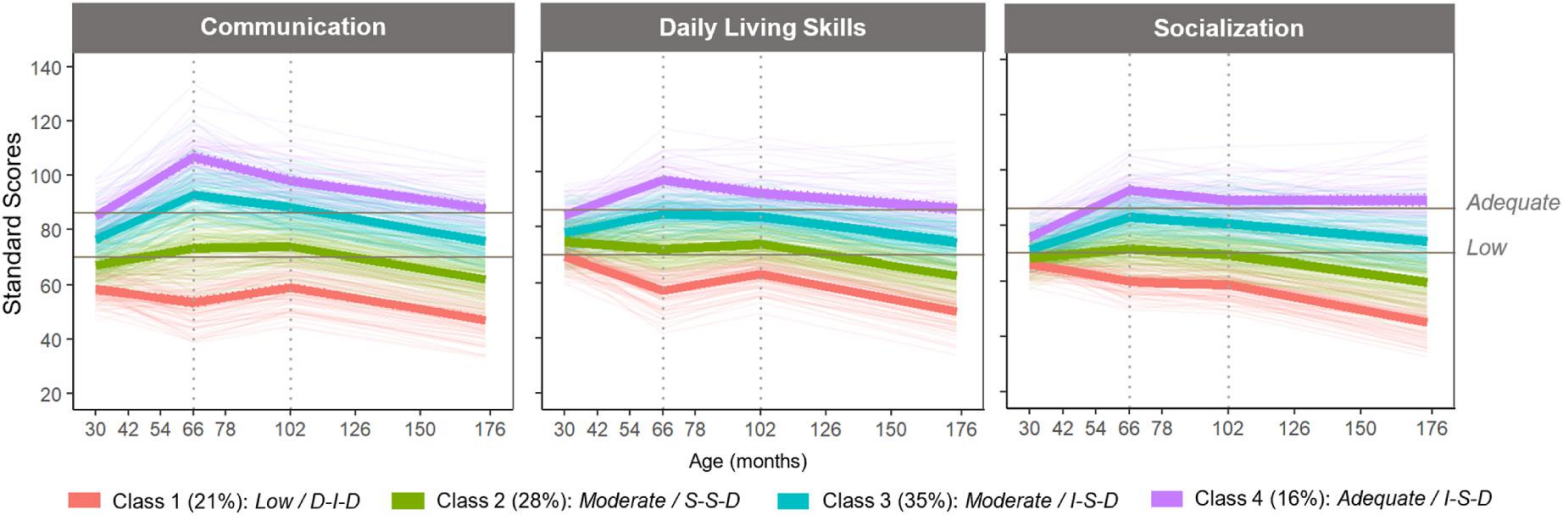
Estimated piecewise trajectories of Vineland Adaptive Behavior Scales (VABS) domains by latent class. The horizontal lines indicate the VABS‐II qualitative descriptors of standard scores (Adequate: 86–114, Moderately Low: 71–85, Low: 20–70). The naming of subgroups is based on the level of adaptive functioning (Low, Moderate, and Adequate; based on VABS‐II descriptors) and the change pattern for each period segmented by the turning points as indicated by the dotted vertical lines (D = Decrease, I = Increase, S = Stable).

The results of parallel‐process LCGA are shown in Table 3. The 4‐class model was selected given the relatively large drops in BIC/SABIC, nearly significant LMR‐LRT statistics compared to the 3‐class model, and an entropy value (0.918) that indicates excellent class distinction. As visualized in Figure 1, the selected model included the following classes characterized by various age‐normed levels of functioning and change rates for the three phases of trajectories (see Table S6 for the latent growth parameter estimates for each latent class):

- Class 1 (C1; *n* = 87, 21%): VABS‐domain standard scores were overall within the low range based on the VABS‐II descriptors (≤70), with decreasing trajectories by age 6, followed by improving trajectories in Communication and DLS up to age 10, and then overall decreasing trajectories after age 10.
- Class 2 (C2; *n* = 113, 28%): VABS‐domain standard scores fell between the moderately low and adequate ranges, with overall stable trajectories by age 10 (except for an improving Communication trajectory between ages 6 and 10), followed by overall decreasing trajectories after age 10.
- Class 3 (C3; *n* = 140, 35%): VABS‐domain standard scores fell between the low and moderately low ranges, with overall improving trajectories by age 6, followed by stable trajectories (except for a decreasing Communication trajectory), and then overall decreasing trajectories after age 10.
- Class 4 (C4; *n* = 66, 16%): VABS‐domain standard scores were overall within the adequate range (>85), with a change pattern similar to Class 3, except for a stable Socialization trajectory after age 10.

**TABLE 3:** Fit statistics for parallel‐process latent class growth analysis (LCGA) models.

# Of class	BIC	SABIC	Entropy	LMR‐LRT	Class proportions (%)
2	40,741.72	40,652.88	0.935	<0.001	45/55
3	39,778.86	39,648.76	0.914	0.030	26/34/40
**4**	**39,232.13**	**39,060.78**	**0.918**	**0.054**	**16/21/28/35**
5	39,100.50	38,887.90	0.893	0.209	14/18/20/20/28
6	39,016.08	38,762.22	0.876	0.660	10/13/18/18/20/21

Abbreviations: BIC, Bayesian information criterion; LMR‐LRT, Lo‐Mendell‐Rubin likelihood ratio test; SABIC, sample size‐adjusted Bayesian information criterion. The selected class solution is bolded.

### Covariates of VABS trajectory class membership

Given the subgroup comparison results in Table 4, the higher adaptive functioning subgroups (C3 and C4) were overall associated with higher NVIQ and a lower level of autism features at diagnosis, higher household income, higher primary caregiver's education, White ethnicity, and Canada‐born status of the primary caregiver as compared to C1 and C2. Moreover, children in C3 were diagnosed later than those in C1 (*b* = 0.05, SE = 0.02, *p* = 0.009). When including all mutually adjusted covariates, *NVIQ at diagnosis* and *household income* survived; the difference in caregiver's education between C3 and C2 also remained significant (*b* = 0.54, SE = 0.26, *p* = 0.040). Interestingly, the differences between C4 and C3 (*b* = −0.07, SE = 0.02, *p* = 0.002) and between C4 and C2 (*b* = 0.05, SE = 0.03, *p* = 0.044) in ages of diagnosis became significant when controlling for other covariates. It is noteworthy that while NVIQ at diagnosis is the strongest covariate, large standard deviations were observed for C3 and C4 (also see Figure S1 for the NVIQ distribution by subgroup).

**TABLE 4:** Covariates of adaptive functioning trajectory class membership.

Covariates	Descriptive statistics by class				Multinomial logistic regression results *b* (SE)					
	C1 (*n* = 87)	C2 (*n* = 113)	C3 (*n* = 140)	C4 (*n* = 66)	C2 versus C1	C3 versus C1	C4 versus C1	C3 versus C2	C4 versus C2	C4 versus C3
Child characteristics at diagnosis										
Male	75 (86%)	92 (81%)	120 (86%)	55 (83%)	−0.39 (0.42)	−0.04 (0.41)	−0.24 (0.47)	0.35 (0.38)	0.15 (0.43)	−0.20 (0.44)
Age of diagnosis (months)	36.01 (8.69)	37.96 (7.84)	39.89 (9.20)	38.92 (8.67)	0.02 (0.02)	0.05 (0.02)**	0.03 (0.02)	0.03 (0.02)	**0.01 (0.02)**	**−0.02 (0.02)**
NVIQ (M‐P‐R cognitive)	34.85 (15.20)	52.36 (16.84)	65.49 (20.65)	82.54 (25.78)	0.08 (0.01)***	0.11 (0.01)***	0.14 (0.02)***	0.04 (0.01)***	0.07 (0.01)***	0.03 (0.01)***
Autism symptom (ADOS‐total CSS)	8.49 (1.51)	7.30 (1.67)	7.38 (1.67)	7.21 (1.70)	−0.52 (0.11)***	−0.47 (0.10)***	−0.53 (0.12)***	0.05 (0.08)	−0.02 (0.10)	−0.06 (0.09)
Family demographic characteristics										
Household income	8.45 (2.72)	8.56 (2.78)	9.44 (2.41)	10.49 (1.27)	0.00 (0.06)	0.15 (0.06)*	0.52 (0.13)***	0.14 (0.06)*	0.37 (0.13)**	0.37 (0.13)**
Primary Caregiver's education										
*High school or less*	8 (9%)	14 (12%)	11 (8%)	2 (3%)	−0.08 (0.23)	0.48 (0.23)*	0.76 (0.28)**	0.56 (0.23)*	0.84 (0.27)**	0.28 (0.28)
*Some post‐secondary education*	41 (47%)	54 (48%)	50 (36%)	23 (35%)						
*Bachelor's degree or higher*	30 (34%)	42 (37%)	75 (54%)	38 (58%)						
Primary Caregiver's ethnicity (1 = White, 0 = Other)										
*White*	51 (59%)	68 (60%)	111 (79%)	51 (77%)	−0.21 (0.34)	0.87 (0.35)*	0.83 (0.43)	1.08 (0.33)**	1.04 (0.40)*	−0.04 (0.43)
*Other*	27 (31%)	40 (35%)	24 (17%)	11 (17%)						
Primary Caregiver's nativity (1 = Canada‐born, 0 = Foreign‐born)										
*Canada‐born*	48 (55%)	69 (61%)	108 (77%)	50 (76%)	0.08 (0.33)	0.95 (0.33)**	0.96 (0.41)*	0.87 (0.33)**	0.89 (0.40)*	0.01 (0.42)
*Foreign‐born*	30 (34%)	39 (35%)	27 (19%)	12 (18%)						

*Note*: *p < 0.05, **p < 0.01, ***p < 0.001; Bolded beta coefficient values represent significant effects (*p* < 0.05) in the adjusted model (also including study site as a covariate).

### Activity participation in adolescence by VABS trajectory class

**FIGURE 2:**
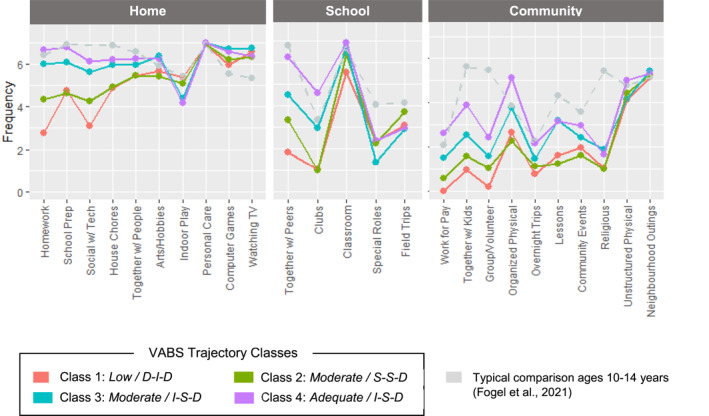
Activity participation in adolescence by Vineland Adaptive Behavior Scales (VABS) trajectory class. Frequency was measured at an 8‐point ordinal scale (0 = never, 1 = once in last 4 months, 2 = few times in last 4 months, 3 = once a month, 4 = few times a month, 5 = once a week, 6 = few times a week, 7 = daily). Activities were ordered according to the magnitude of group differences from the largest (left) to smallest (right).

Significant differences across the four VABS trajectory classes were observed in the participation frequency of 17 out of 25 types of activities during adolescence (see Table S7 for detailed group comparison results). As shown in Figure 2, the activity types with the largest group differences (χ^2^(3) > 30, all *p* < 0.001) for each setting are as follows: *homework* (*χ* ^2^ = 83.24), *school preparation* (*χ* ^2^ = 39.34), and *socializing using technology* (*χ* ^2^ = 37.08) at home; *together with peers* (*χ* ^2^ = 88.05) and *clubs* (*χ* ^2^ = 62.60) at school; *working for pay* (*χ* ^2^ = 63.12), *together with other children* (*χ* ^2^ = 49.22), *group/volunteer activities* (*χ* ^2^ = 45.23), and *organized physical activities* (*χ* ^2^ = 36.00) in community. Those in C3 and C4 showed more active participation in productive/non‐leisure or interactive home activities (e.g., doing homework, helping with household chores, socializing using technology). Particularly, those in C4 showed more frequent participation in activities that involve more social interaction or functional skills (e.g., together with peers, school clubs, group/volunteer activities, working for pay) across settings.

## DISCUSSION

In this study we tracked the multivariate trajectories of adaptive functioning in an inception cohort from autism diagnosis in early childhood to early adolescence. Our findings revealed pronounced chronogeneity (i.e., large variability in both levels and change rates) of adaptive functioning across developmental stages. Importantly, we identified two turning points that respectively represent transitions into school and early adolescence. Before the first turning point at age 5‐6, overall marked progress was observed in social communication skills relative to age norms; however, such improvement seemed to decelerate during school age, with the rates of decrease becoming greater into adolescence. In contrast, children's daily living skills remained relatively stable until around age 10. These observations are generally consistent with the previous reports of quadratic change over a longer‐term follow‐up to adolescence or adulthood, in which early progress was followed by decreasing slopes of adaptive functioning development from around ages 6–10 (Baghdadli et al., 2018; Bal et al., 2015; Lord et al., 2015; Meyer et al., 2018).

School transitions often pose additional challenges and barriers to further improvement in adaptive functioning among Autistic children (Fontil et al., 2020; Nuske et al., 2019). The unpredictable environment and the need for acquiring a new set of skills may expose them to heightened stress and anxiety and may complicate the generalization of their acquired skills. The identification of a second turning point toward the end of elementary school years associated with further decelerating growth in adaptive functioning during adolescence highlights the importance of *continuous* supports for Autistic people's changing needs over time (Kurth & Mastergeorge, 2010; Mandy et al., 2016).

It is important to note that the *average* nonlinear trends of adaptive functioning development are insufficient to describe the longitudinal heterogeneity observed in our sample. We further parsed four subgroups that not only differed in levels of adaptive functioning and rates of change across the three phases of trajectories in certain VABS domains, but also in several individual and family characteristics. Specifically, a subgroup of children (C1, 21%) was characterized by a distinct pattern from other subgroups across childhood: VABS standard scores within the low range (≤70) and early decreases across VABS domains relative to age norms, followed by late improvement in communication and daily living skills from around ages 6–10. Previous research has also reported a subgroup, with an approximate prevalence of 20%, that shows regressions in language and adaptive functioning during infancy and preschool age (Pickles et al., 2022; Sacrey et al., 2019). However, the observed improving trends during early school years indicate that some catch‐up in communication and daily living skills seemed to “kick in” for this subgroup. This may be explained by their relatively elevated autism features and cognitive delay at diagnosis, which are often associated with more difficulties in acquiring age‐appropriate functional skills during early childhood (Hill et al., 2015; Kanne et al., 2011). In addition, the lower family socioeconomic status (SES) observed in this subgroup may be a potential barrier to early access to services for supporting children's adaptive functioning development. Although we did not evaluate service use, structural barriers to services and unmet needs in low‐SES families with Autistic children have been reported across the literature (Pickard & Ingersoll, 2016; Smith et al., 2020). Transitioning into school may ensure children from low‐SES families receive services within school settings, where the SES‐related disparities in access to service tend to diminish (Suhrheinrich et al., 2021), thus potentially contributing to improvement at school age in this subgroup of children.

By contrast, 16% of our sample were classified into the highest adaptive functioning group (C4) with the most marked progress during preschool age across domains. Although decreasing slopes were observed after the first turning point, their level of functioning remained within the adequate range (standard scores >85). A notable characteristic of this subgroup was that their *social* functioning remained stable after transitioning into adolescence in contrast to the overall decreasing pattern during adolescence observed in other domains and other subgroups. This finding resonates with the previous reports of increasing heterogeneity in social outcomes over time as demonstrated by at least a subpopulation of Autistic adolescents with strengths in social and relational skills (Cost et al., 2021; Warren et al., 2021). Children classified into C4 tended to have higher cognitive ability at diagnosis, although with significant variability, as around one‐third of them had NVIQ scores below 70. However, at the adolescent follow‐up, all retained participants in this subgroup had NVIQ scores of 70 or higher (see Figure S1), indicating a narrowing gap between cognitive and adaptive functioning over time. Conversely, the variability of NVIQ scores appeared wider at adolescence for the lower adaptive functioning groups. Specifically, all children in C1 had NVIQ scores below 70 at diagnosis, while approximately 18% of them had an NVIQ ≥70 by adolescence. These observations suggest that the gap between cognitive and adaptive skills varies across the Autism spectrum and across developmental stages, thus warranting comprehensive and continuous outcome assessments to better capture the variable functioning profiles of Autistic people (Alvares et al., 2020; Georgiades & Kasari, 2018). Further longitudinal research incorporating consistent measures and data collection schedules between cognitive and adaptive functioning is warranted to gain deeper insights into the co‐developmental patterns of these domains over time.

Moreover, children in C4 tended to come from higher‐income families, and their caregivers were more likely to be Canadian‐born and more educated than caregivers of children in other subgroups. Our finding that family income and caregiver's education were significant correlates of child's adaptive functioning development may be explained by their associations with more access to and knowledge about resources and services (Pickard & Ingersoll, 2016; Smith et al., 2020) and greater capacity for advocacy (Lord et al., 2020). Further, the observed positive effect of primary caregivers' nativity on the child's adaptive functioning may reflect the previously documented disparities in navigating health systems and social support among immigrant caregivers with Autistic children in North America due to language barriers, cultural differences, and limited social networks (Khanlou et al., 2017; Lim et al., 2021). These findings regarding family demographics highlight potential inequities in access to diagnostic services and care across diverse populations (Aylward et al., 2021; Shenouda et al., 2023), which requires system change to better support marginalized families in supporting their children's functional development.

The other two subgroups with adaptive functioning between low and adequate levels (C2 and C3; 63% of our sample) are overall characterized by child and family covariates that were similar to those for C1 and C4, respectively. For instance, both C1 and C2 were diagnosed slightly earlier potentially due to their cognitive delay and lower adaptive functioning levels, and shared several family demographic characteristics (e.g., relatively lower household income, and less educated caregivers, non‐White races, and non‐Canada‐born status), while C3 and C4 were toward the opposite (i.e., later age of diagnosis and higher SES status). However, children classified to C2, which was characterized by generally stable but lower‐level adaptive functioning trajectories, did not differ from the higher‐functioning groups in autism features at diagnosis. Notably, children in C3 on average were diagnosed *later* than their neighboring subgroups (C2 and C4) when controlling for other characteristics. This indicates that given the similar level of autism features, cognitive ability at diagnosis, and family characteristics, they tended to be later diagnosed and thus might have later access to services. The delay in diagnosis may prevent them from reaching their full potential for more adaptive growth during preschool age as observed in C4. Thus, this finding highlights the importance of *timely* diagnosis and access to services for promoting more adaptive developmental pathways (Elsabbagh, 2020; Lord et al., 2022). It also highlights the pressing issue of long waitlists for needs‐based autism services, even with diagnostic evaluation available through universal healthcare in Canada (Das et al., 2022; Tsiplova et al., 2023). Addressing this challenge is crucial to ensure that all families receive the necessary support right after diagnosis, allowing their children to reach their full developmental potential during the early years.

In the current study we also assessed daily activity participation in adolescence as a meaningful distal outcome associated with adaptive functioning trajectories. The significant differences observed across VABS trajectory subgroups in many types of activities across home, school, and community settings reflect the close relation between the development of adaptive functioning and later participation outcomes. Particularly, larger group differences were observed in activities that require more adaptive functioning skills (e.g., working for pay, doing homework, helping with household chores) and social interactions (e.g., socializing using technology, school clubs, together with peers, community gatherings). Autistic adolescents in C4 tended to show comparable levels of home participation and social participation at school to typically developing peers as reported by other studies (Egilson et al., 2018; Fogel et al., 2021). These findings indicate that at least a subpopulation of Autistic people who follow more adaptive pathways actively participate in a variety of daily activities across settings in adolescence, which may be connected to their functional independence and successful transitions to adulthood (Myers et al., 2015; Weiss et al., 2021). However, additional support or adaptation may be needed in less structured or unfamiliar settings (e.g., community) to ensure person‐environment fit that potentiates Autistic people's participation in various aspects of life, considering their individual strengths and needs (Chen et al., 2023; Lai et al., 2020; Szatmari et al., 2021).

Despite these important and novel findings as derived from a large inception cohort with long‐term follow‐up, some caveats need to be considered. First, given the use of standard scores, the current findings should be interpreted as norm‐referenced instead of criterion‐related functional change over time (Sullivan et al., 2014). The computation of longitudinally comparable scores was challenging based on the VABS‐II raw items that were subject to the basal/ceiling rule. However, the availability of person ability scores in the new version (VABS‐3) may be a promising alternative in the future investigation of change in adaptive functioning based on criterion‐referenced or mastery‐oriented measures (Farmer et al., 2023). Second, significant attrition over time, which is a universal challenge to longitudinal research may add to the uncertainty of estimations (i.e., larger standard errors) despite our application of FIML and sensitivity analysis to cope with missingness. Although the overall missing pattern in this study appears unrelated to child and family characteristics, future longitudinal studies may benefit from oversampling subgroups that are susceptible to dropouts to ensure representativeness of the diverse population. Female and non‐White participants were also underrepresented in our sample, who should be recruited more effectively to ensure the generalizability of the findings in future studies. Further, the varying schedules of data collection across measures made it challenging to examine time‐varying covariate effects that allow for a better understanding of the temporal dynamics across multiple behavioral domains. Future research should also aim to collect detailed information on intervention characteristics, including the types of intervention, their intensity, duration, and fidelity (Tsiplova et al., 2023). This will allow researchers to identify specific intervention factors that have the most significant impact on fostering adaptive outcomes and potentially even “shaping” the trajectories toward a “doing well” outcome.

### Conclusion

In this study, we took a fine‐grained approach to characterize the chronogeneity of adaptive functioning and its associated factors from early childhood to adolescence among an inception cohort of Autistic people. Four clinically meaningful subgroups were identified that not only vary by their level of adaptive functioning but by the rate of change for certain adaptive functioning domains and developmental periods segmented by turning points that indicate transitions into school age and emerging adolescence. Each subgroup was also associated with a variety of individual and family characteristics, with nonverbal IQ at diagnosis and household income identified as the strongest correlates of children's development of adaptive functioning. Specifically, we identified a subgroup characterized by an overall adequate level of adaptive functioning, notable growth over early childhood across domains, and more active daily activity participation outcomes with social strengths in adolescence, as empirical support of “doing well” (Szatmari et al., 2021) and progress over time in meaningful outcome domains (Georgiades & Kasari, 2018). The observed differences in daily participation across subgroups underscore the importance of providing additional support in specific areas of life given individual levels of functioning. It also emphasizes the need to adopt varying metrics of “doing well” to accommodate the unique strengths and needs of each Autistic individual. Taken together, our findings highlight the significance of studying autism heterogeneity from developmental and multidimensional perspectives to inform timely and tailored supports for Autistic people.

## AUTHOR CONTRIBUTIONS

**Yun‐Ju Chen:** Conceptualization; Data curation; Formal analysis; Methodology; Software; Visualization; Writing – original draft; Writing – review & editing. **Eric Duku:** Investigation; Methodology; Supervision; Writing – review & editing. **Peter Szatmari:** Funding acquisition; Investigation; Resources; Supervision; Writing – review & editing. **Mackenzie Salt:** Writing – review & editing. **Isabel Smith:** Funding acquisition; Investigation; Writing – review & editing. **Annie Richard:** Writing – review & editing. **Lonnie Zwaigenbaum:** Funding acquisition; Investigation; Writing – review & editing. **Tracy Vaillancourt:** Funding acquisition; Investigation; Writing – review & editing. **Anat Zaidman‐Zait:** Investigation; Writing – review & editing. **Stelios Georgiades:** Conceptualization; Funding acquisition; Investigation; Supervision; Writing – review & editing.

## CONFLICT OF INTEREST STATEMENT

Dr. Peter Szatmari has received royalties from Guilford Press and Simon & Schuster. The remaining authors declare that they have no competing or potential conflicts of interest.

## ETHICAL CONSIDERATIONS

The Pathways in ASD study was approved by the local research ethics boards at all recruitment sites.

## Supporting information

Supplementary Material

## Data Availability

The data that supports the findings of this study are available from the corresponding author upon reasonable request.
