# Supplementary material for: Trajectories of adaptive functioning from early childhood to adolescence in autism: Identifying turning points and key correlates of chronogeneity
Source: JCPP Adv. 2023 Dec 14;4(2):e12212. doi: 10.1002/jcv2.12212 (PMC11143958; doi:10.1002/jcv2.12212)
Supplement: Supplementary file 1 — Supplementary Material [file JCV2-4-e12212-s001.docx]

**Supporting Information**

Table S1. Repeated measures across visits of the *Pathways in ASD* study

| **Visit** | **1^*^** | **2^*^** | **3^*^** | **4** | **5** | **6** | **7** | **8** | **9** | **10** |
| --- | --- | --- | --- | --- | --- | --- | --- | --- | --- | --- |
| Age Range (yrs) | 2-5 | 2.5-5.5 | 3-6 | 5.5-7.5 | 7.5-8 | 8-9.5 | 9.5-10 | 10-12 | 12-14 | 14-17 |
| VABS | x | (x) | x | x |  | x |  | x | x^†^ |  |
| FBIQ | x | x | x |  | x | x | x | x | x |  |
| PEM-CY |  |  |  |  |  |  |  |  | x | x |

*: Accelerated longitudinal design (various age at study enrollment); †: Age range was 12-17 years of age for the VABS; (x): Data were collected but not used or merged to the neighboring age bands for more balanced age spacing in the current analysis. FBIQ= Family Background Information Questionnaire.

Table S2. Descriptive statistics of VABS data restructured by chronological age (N = 406)

| **Study Visits** | **Visits 1 & 3** | | | | **Visit 4** | **Visit 6** | **Visit 8** | **Visit 9** | |
| --- | --- | --- | --- | --- | --- | --- | --- | --- | --- |
| **Time Points by Age** | **T1** | **T2** | **T3** | **T4** | **T5** | **T6** | **T7** | **T8** | **T9** |
| Age Interval (months) | 24-36 | 36-48 | 48-60 | 60-72 | 72-96 | 96-120 | 120-144 | 144-168 | ≥168 |
| Age Mean (SD) | 31.1 (3.4) | 42.3 (3.3) | 53.6 (3.5) | 66.3 (3.9) | 79.3 (3.8) | 104.9 (2.7) | 129.1 (3.0) | 158.5 (7.2) | 176.3 (7.2) |
| N | 148 | 284 | 243 | 110 | 320 | 269 | 220 | 93 | 108 |
| VABS Domain Standard Scores Mean (SD) | | | | | | | | | |
| Communication (COM) | 71.0 (12.0) | 73.6 (17.9) | 79.3 (19.0) | 83.3 (17.9) | 82.3 (19.4) | 80.8 (16.0) | 78.2 (17.1) | 71.8 (17.0) | 68.6 (16.0) |
| Daily Living Skills (DLS) | 76.8 (10.9) | 75.8 (12.8) | 77.4 (12.9) | 81.5 (13.9) | 78.4 (15.2) | 78.9 (12.2) | 75.6 (14.0) | 71.2 (15.0) | 68.1 (15.3) |
| Socialization (SOC) | 73.9 (8.5) | 72.3 (9.8) | 73.6 (12.0) | 77.0 (12.1) | 76.7 (13.5) | 75.2 (13.1) | 72.3 (15.8) | 68.7 (18.2) | 67.2 (16.6) |

Table S3. Sensitivity analysis of latent growth parameter estimates under different data conditions

|  | COM | | | | DLS | | | | SOC | | | |
| --- | --- | --- | --- | --- | --- | --- | --- | --- | --- | --- | --- | --- |
|  | INT | SLP_1_ | SLP_2_ | SLP_3_ | INT | SLP_1_ | SLP_2_ | SLP_3_ | INT | SLP_1_ | SLP_2_ | SLP_3_ |
| Diff_median | 1.33 | -.56 | .19 | .00 | .49 | -.21 | .10 | .01 | .34 | -.08 | -.11 | .03 |
| SE_median | 6.08 | 2.32 | 1.57 | .70 | 4.71 | 1.25 | 1.11 | .69 | 4.90 | 2.12 | 1.42 | .93 |

Samples with ≥ 1 (N = 406) vs. ≥ 5 (N = 231) out of 9 data points of VABS

Note. Diff_median=median differences between individual estimates derived from samples with the two data conditions; SE_median=median standard errors of individual estimates from sample with ≥1 time-point(s); INT=intercept; SLP=slope. The absolute values of median absolute differences of individual latent growth parameter estimates derived from samples with the two data conditions were all much smaller than the median standard errors of estimates, indicating no salient differences across growth parameter estimates between samples with different data conditions.

| Model | χ^2^ | df | CFI | TLI | RMSEA [95% CI] |
| --- | --- | --- | --- | --- | --- |
| COM (univariate) | | | | | |
| Linear | 476.07 | 39 | .773 | .796 | .166 [.153, .180] |
| Quadratic | 232.42 | 35 | .897 | .897 | .118 [.104, .132] |
| Piecewise (T4 knot) | 161.40 | 35 | .934 | .934 | .094 [.080, .109] |
| Piecewise (T4, T6 knots) | **76.42** | **30** | **.976** | **.972** | **.062 [.045, .079]** |
| DLS (univariate) | | | | | |
| Linear | 264.54 | 39 | .838 | .854 | .119 [.106, .133] |
| Quadratic | 145.38 | 35 | .921 | .921 | .088 [.074, .103] |
| Piecewise (T4 knot) | 136.42 | 35 | .927 | .927 | .084 [.070, .100] |
| Piecewise (T4, T6 knots) | **48.55** | **30** | **.987** | **.984** | **.039 [.017, .058]** |
| SOC (univariate) | | | | | |
| Linear | 289.50 | 39 | .810 | .829 | .126 [.112, .140] |
| Quadratic | 117.26 | 35 | .937 | .937 | .076 [.061, .092] |
| Piecewise (T4 knot) | 69.58 | 35 | .974 | .974 | .049 [.032, .066] |
| Piecewise (T4, T6 knots) | **48.91** | **30** | **.986** | **.983** | **.039 [.017, .059]** |
| COM+DLS+SOC (multivariate/parallel-process) | | | | | |
| Piecewise (T4, T6 knots) | **485.43** | **267** | **.968** | **.959** | **.045 [.038, .051]** |

Table S4. Fit statistics of latent growth curve models (LGMs)

Table S5. Latent growth factor associations in parallel-process LGM

|  | **COM** | | | | **DLS** | | | | **SOC** | | | |
| --- | --- | --- | --- | --- | --- | --- | --- | --- | --- | --- | --- | --- |
|  | INT | SLP_1_ | SLP_2_ | SLP_3_ | INT | SLP_1_ | SLP_2_ | SLP_3_ | INT | SLP_1_ | SLP_2_ | SLP_3_ |
| INT  **COM** | -- |  |  |  |  |  |  |  |  |  |  |  |
| SLP_1_ | .24^*^ | -- |  |  |  |  |  |  |  |  |  |  |
| SLP_2_ | -.69^***^ | -.45^***^ | -- |  |  |  |  |  |  |  |  |  |
| SLP_3_ | .15 | -.21 | -.13 | -- |  |  |  |  |  |  |  |  |
| INT  **DLS** | .65^***^ | .12 | -.40^**^ | .04 | -- |  |  |  |  |  |  |  |
| SLP_1_ | .45^***^ | .85^***^ | -.46^***^ | .05 | .09 | -- |  |  |  |  |  |  |
| SLP_2_ | -.65^***^ | -.31^*^ | .72^***^ | -.39^*^ | -.63^**^ | -.38^**^ | -- |  |  |  |  |  |
| SLP_3_ | .30^*^ | .18 | -.26 | .88^***^ | .06 | .43^**^ | -.50^**^ | -- |  |  |  |  |
| INT | .66^***^ | -.13 | -.30^*^ | .18 | .80^***^ | -.16 | -.24 | .26 | -- |  |  |  |
| SLP_1_  **SOC** | .35^**^ | .86^***^ | -.50^***^ | -.06 | .09 | .95^***^ | -.58^***^ | .21 | -.17 | -- |  |  |
| SLP_2_ | -.22 | -.13 | .76^***^ | -.22 | -.30 | .00 | .64^***^ | -.04 | -.16 | -.17 | -- |  |
| SLP_3_ | .39^***^ | .24^*^ | -.40^**^ | .58^***^ | .30^*^ | .30^*^ | -.39^**^ | .68^***^ | .36^**^ | .16 | -.22 | -- |

Cross-domain correlations for the corresponding growth parameter; ^*^*p* < .05, ^**^*p* < .01, ^***^*p* < .001

Table S6. Latent growth parameter estimates (unstandardized) by latent class

*Class 1 (n = 87): Low / decrease – increase (SOC stable) – decrease*

|  | COM | | | DLS | | | SOC | | |
| --- | --- | --- | --- | --- | --- | --- | --- | --- | --- |
|  | Mean | SE | *p* | Mean | SE | *p* | Mean | SE | *p* |
| INT | 58.31 | .95 | <.001 | 67.73 | .98 | <.001 | 67.57 | .81 | <.001 |
| SLP_1_ | -2.88 | .58 | <.001 | -3.85 | .58 | <.001 | -3.17 | .42 | <.001 |
| SLP_2_ | 2.72 | .47 | <.001 | 2.05 | .40 | <.001 | -.61 | .39 | .112 |
| SLP_3_ | -2.09 | .17 | <.001 | -2.39 | .22 | <.001 | -2.19 | .17 | <.001 |

|  | COM | | | DLS | | | SOC | | |
| --- | --- | --- | --- | --- | --- | --- | --- | --- | --- |
|  | Mean | SE | *p* | Mean | SE | *p* | Mean | SE | *p* |
| INT | 65.39 | 1.34 | <.001 | 74.71 | 1.49 | <.001 | 70.16 | 1.26 | <.001 |
| SLP_1_ | 2.97 | .72 | <.001 | -.50 | .71 | .480 | -.26 | .65 | .687 |
| SLP_2_ | -.17 | .40 | .669 | -.16 | .36 | .667 | .13 | .38 | .735 |
| SLP_3_ | -1.89 | .25 | <.001 | -1.87 | .21 | <.001 | -1.96 | .23 | <.001 |

*Class 2 (n = 113): Moderate / stable (COM increase) – stable – decrease*

*Class 3 (n = 140): Moderate / increase – stable (COM decrease) – decrease*

|  | COM | | | DLS | | | SOC | | |
| --- | --- | --- | --- | --- | --- | --- | --- | --- | --- |
|  | Mean | SE | *p* | Mean | SE | *p* | Mean | SE | *p* |
| INT | 77.50 | 1.88 | <.001 | 77.54 | 1.39 | <.001 | 73.93 | 1.26 | <.001 |
| SLP_1_ | 5.09 | .58 | <.001 | 2.47 | .55 | <.001 | 2.64 | .50 | <.001 |
| SLP_2_ | -1.15 | .44 | .008 | -.39 | .32 | .221 | .03 | .38 | .934 |
| SLP_3_ | -2.17 | .19 | <.001 | -1.51 | .19 | <.001 | -1.31 | .37 | <.001 |

*Class 4 (n = 66): Adequate / increase – stable (COM decrease) – decrease (SOC stable)*

|  | COM | | | DLS | | | SOC | | |
| --- | --- | --- | --- | --- | --- | --- | --- | --- | --- |
|  | Mean | SE | *p* | Mean | SE | *p* | Mean | SE | *p* |
| INT | 87.37 | 1.90 | <.001 | 87.78 | 2.17 | <.001 | 80.42 | 1.43 | <.001 |
| SLP_1_ | 6.61 | .80 | <.001 | 3.08 | .78 | .001 | 3.64 | .63 | <.001 |
| SLP_2_ | -2.65 | .60 | <.001 | -1.01 | .60 | .094 | -.69 | .59 | .242 |
| SLP_3_ | -1.56 | .32 | <.001 | -1.06 | .39 | .007 | -.40 | .44 | .358 |

Table S7. PEM-CY activity participation frequency in adolescence by VABS trajectory class

|  | C1  (n=40) | C2  (n=43) | C3 (n=68) | C4  (n=35) | Group Comparison χ^2^ | |
| --- | --- | --- | --- | --- | --- | --- |
|  |  |  |  |  | **Overall**  (df=3) | **Pairwise^†^** |
|  | Mean (SE) | | | |  |  |
| Home |  |  |  |  |  |  |
| Computer games | 5.97 (.37) | 6.19 (.31) | 6.71 (.15) | 6.59 (.21) | 5.03 | -- |
| Indoor play | 5.37 (.33) | 5.08 (.38) | 4.39 (.29) | 4.18 (.37) | 8.48^*^ | 1>3^*^, 1>4^*^ |
| Arts/hobbies | 5.68 (.33) | 5.42 (.33) | 6.37 (.14) | 6.25 (.23) | 9.68^*^ | 3>2^**^, 4>2^*^ |
| Watching TV | 6.58 (.21) | 6.35 (.26) | 6.73 (.11) | 6.40 (.16) | 3.49 | -- |
| Together w/ people | 5.48 (.27) | 5.47 (.25) | 5.95 (.18) | 6.26 (.17) | 10.74^*^ | 4>1^*^, 4>2^**^ |
| Social media | 3.10 (.53) | 4.27 (.45) | 5.64 (.28) | 6.11 (.22) | 37.08^***^ | **3>1^***^**, 3>2^*^, **4>1^***^**, **4>2^***^** |
| House chores | 4.90 (.41) | 4.95 (.38) | 5.97 (.17) | 6.23 (.21) | 15.34^**^ | 3>1^*^, 3>2^*^, **4>1^**^**, **4>2^**^** |
| Personal care | 6.90 (.06) | 6.90 (.06) | 6.99 (.01) | 7.00 (.00) | 6.72 | -- |
| School preparation | 4.78 (.47) | 4.66 (.48) | 6.10 (.23) | 6.77 (.11) | 39.34^***^ | 3>1^*^, 3>2^**^, **4>1^***^**, **4>2^***^**, 4>3^*^ |
| Homework | 2.79 (.51) | 4.34 (.46) | 5.99 (.22) | 6.65 (.10) | 83.24^***^ | 2>1^*^, **3>1^***^**, **3>2^**^**, **4>1^***^**, **4>2^***^**, 4>3^*^ |
| School |  |  |  |  |  |  |
| Classroom | 5.53 (.42) | 6.38 (.24) | 6.64 (.12) | 6.92 (.05) | 18.94^***^ | 3>1^*^, **4>1^**^**, 4>2^*^, 4>3^*^ |
| Field trips/events | 3.06 (.33) | 3.70 (.31) | 2.91 (.19) | 2.94 (.26) | 4.75 | 2>3^*^ |
| School clubs | 1.03 (.32) | .99 (.34) | 2.95 (.36) | 4.59 (.44) | 62.60^***^ | **3>1^***^**, **3>2^***^**, **4>1^***^**, **4>2^***^**, **4>3^**^** |
| Together w/ peers | 1.84 (.44) | 3.33 (.51) | 4.49 (.33) | 6.25 (.26) | 88.05^***^ | 2>1^*^, **3>1^***^**, **4>1^***^**, **4>2^***^**, **4>3^***^** |
| Special roles | 2.31 (.50) | 2.24 (.46) | 1.37 (.30) | 2.38 (.51) | 4.97 | -- |
| Community |  |  |  |  |  |  |
| Neighborhood outings | 5.15 (.27) | 5.26 (.21) | 5.43 (.13) | 5.31 (.20) | 1.17 | -- |
| Community events | 1.98 (.27) | 1.64 (.23) | 2.43 (.18) | 2.96 (.26) | 16.81^**^ | 3>2^**^, 4>1^*^, **4>2^***^** |
| Organized physical | 2.68 (.37) | 2.29 (.42) | 3.77 (.31) | 5.14 (.35) | 36.00^***^ | 3>1^*^, **3>2^**^**, **4>1^***^**, **4>2^***^**, **4>3^**^** |
| Unstructured physical | 4.12 (.34) | 4.43 (.28) | 4.18 (.24) | 5.03 (.30) | 5.71 | 4>1^*^, 4>3^*^ |
| Lessons | 1.63 (.41) | 1.24 (.36) | 3.21 (.32) | 3.19 (.45) | 23.35^***^ | **3>1^**^**, **3>2^***^**, 4>1^*^, **4>2^**^** |
| Group/volunteer | .21 (.12) | 1.04 (.31) | 1.59 (.28) | 2.44 (.44) | 45.23^***^ | 2>1^*^, **3>1^***^**, **4>1^***^**, 4>2^**^ |
| Religious | 1.05 (.32) | 1.02 (.29) | 1.88 (.28) | 1.65 (.37) | 6.28 | 3>1^*^, 3>2^*^ |
| Together w/ other kids | .96 (.30) | 1.59 (.28) | 2.55 (.26) | 3.89 (.35) | 49.22^***^ | **3>1^***^**, 3>2^*^, **4>1^***^**, **4>2^***^**, **4>3^**^** |
| Working for pay | .01 (.03) | .59 (.29) | 1.51 (.29) | 2.63 (.49) | 63.12^***^ | **3>1^***^**, 3>2^*^, **4>1^***^**, **4>2^***^** |
| Overnight trips | .79 (.17) | 1.14 (.20) | 1.47 (.17) | 2.15 (.24) | 23.90^***^ | **3>1^**^**, **4>1^***^**, **4>2^**^**, 4>3^*^ |

^*^*p* < .05, ^**^*p* < .01, ^***^*p* < .001 (BCH 3-step multinomial logistic regression tests). ^†^Nonsignificant pairs of group comparison are not shown; those with Bonferroni-corrected *p*<.008 were bolded.

Frequency was measured at an 8-point ordinal scale (0=never, 1=once in last 4 months, 2=few times in last 4 months, 3=once a month, 4=few times a month, 5=once a week, 6=few times a week, 7=daily).

Figure S1. Distribution of NVIQ by adaptive functioning trajectory subgroup at diagnosis (T1) versus at adolescence (T9)


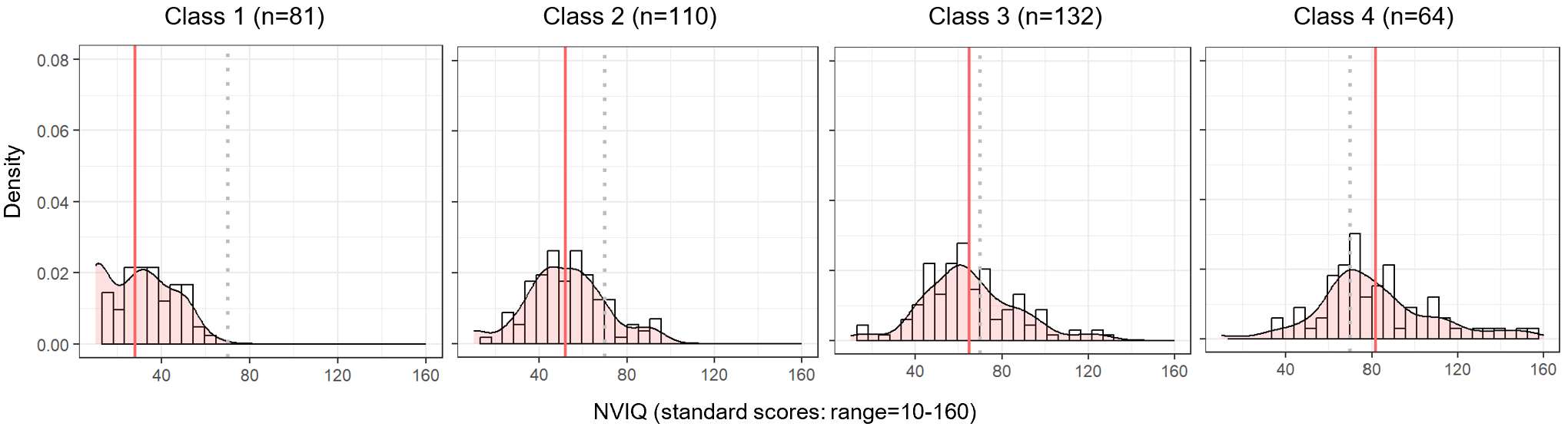
Nonverbal IQ at diagnosis (M-P-R cognitive subscale)

Nonverbal IQ at adolescence (WASI-II/Leiter-R/Leiter-3)


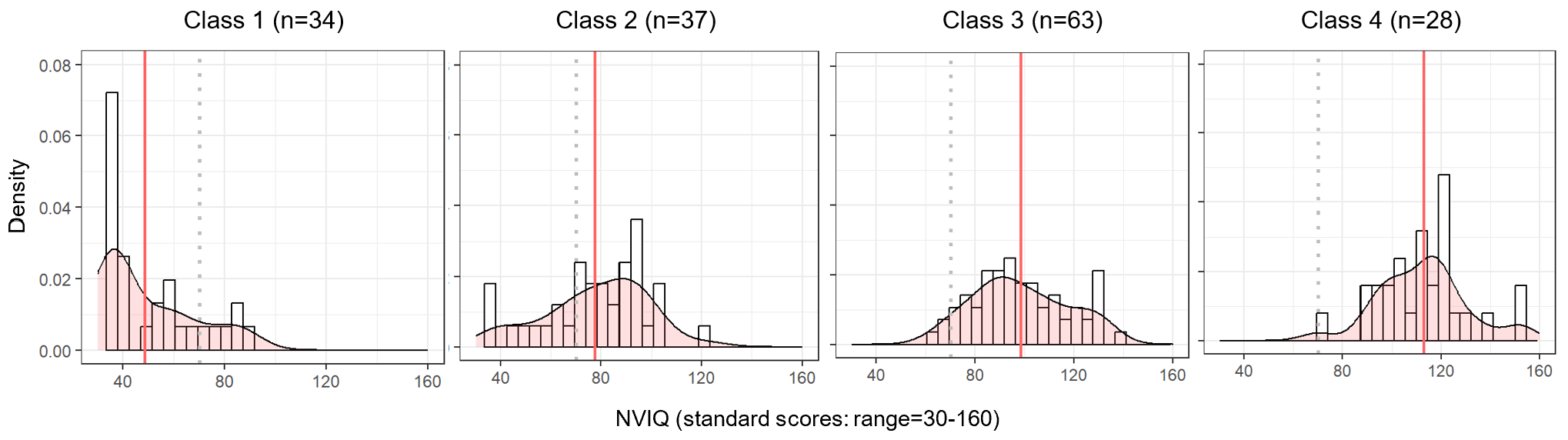


Note. The red vertical lines indicate the mean NVIQ (corrected for classification uncertainty), and the dotted vertical grey lines represent the NVIQ=70 reference line.
